# Endoscopic ultrasound-guided drainage for infected biloma using a unique long-type balloon catheter

**DOI:** 10.1055/a-2340-8713

**Published:** 2024-07-03

**Authors:** Yuichi Takano, Naoki Tamai, Jun Noda, Tetsushi Azami, Fumitaka Niiya, Fumiya Nishimoto, Masatsugu Nagahama

**Affiliations:** 1 26858 Division of Gastroenterology, Department of Internal Medicine, Showa University Fujigaoka Hospital, Yokohama, Japan

Endoscopic ultrasound (EUS)-guided drainage is a widely performed procedure. However, tract dilation can sometimes present challenges and many dedicated devices have been developed 11 22 33 . Herein, we describe a case of successful tract dilation using a unique long-type balloon catheter ( Fig. 1Fig. 1 ).

**Fig. 1:**
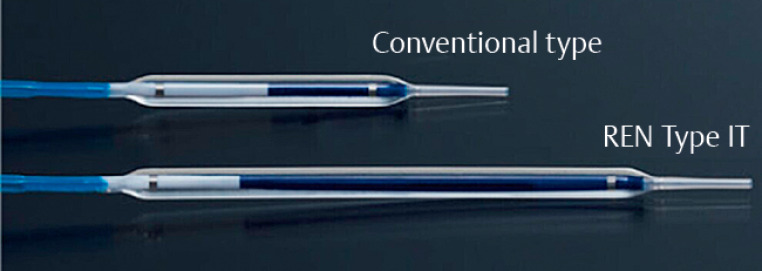
**Fig. 1** A unique long-type balloon catheter of 3 mm in diameter and 6 cm in length (REN Type IT; Kaneka). This balloon is twice as long as the 3 cm-length conventional type (REN, Kaneka).

A 77-year-old man who underwent open extended right hepatectomy, extrahepatic bile duct resection, and choledochojejunostomy for gallbladder cancer developed abdominal pain and fever 15 days postoperatively. Contrast-enhanced computed tomography revealed fluid collection under the right diaphragm, suggesting an infected biloma. Due to the position of the ascending colon in the puncture line, percutaneous drainage was not possible ( Fig. 2Fig. 2 ). As a result, EUS-guided drainage was selected.

**Fig. 2:**
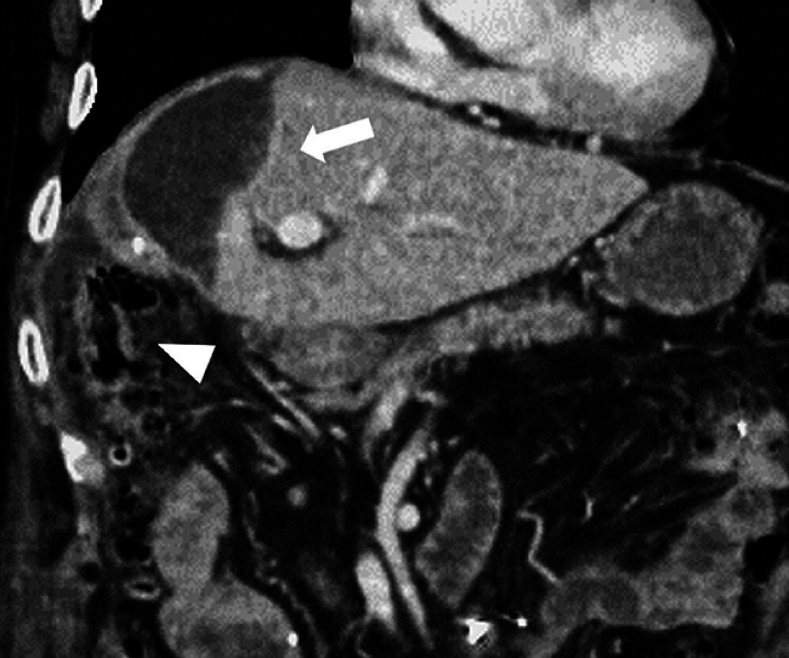
**Fig. 2** Contrast-enhanced computed tomography demonstrates fluid collection under the right diaphragm, suggesting an infected biloma (arrow). The ascending colon is present in the puncture line (arrowhead), making percutaneous drainage impossible.

An echoendoscope (GF-UCT260; Olympus Medical Systems, Tokyo, Japan) was inserted into the duodenal bulb and twisted counterclockwise, revealing a biloma measuring 58 × 43 mm. The distance from the echoendoscope to the biloma was 46 mm. A 19G needle (EZshot3, Olympus Medical Systems) was used to puncture through the liver parenchyma. After injecting contrast medium, a 0.025-inch guidewire (Visiglide2, Olympus Medical Systems) was placed. A balloon catheter with a diameter of 3 mm and length of 6 cm (REN Type IT; Kaneka, Tokyo, Japan) was inserted smoothly, and tract dilation was performed. The expanded balloon was clearly visible on the fluoroscopic image ( Fig. 3Fig. 3 ). Finally, a 6-Fr pigtail-type endoscopic nasobiliary drainage (NB-Braid; Piolax, Yokohama, Japan) was placed, and the procedure was completed without any adverse events ( Fig. 4Fig. 4 , Video 1Video 1 ).

**Fig. 3:**
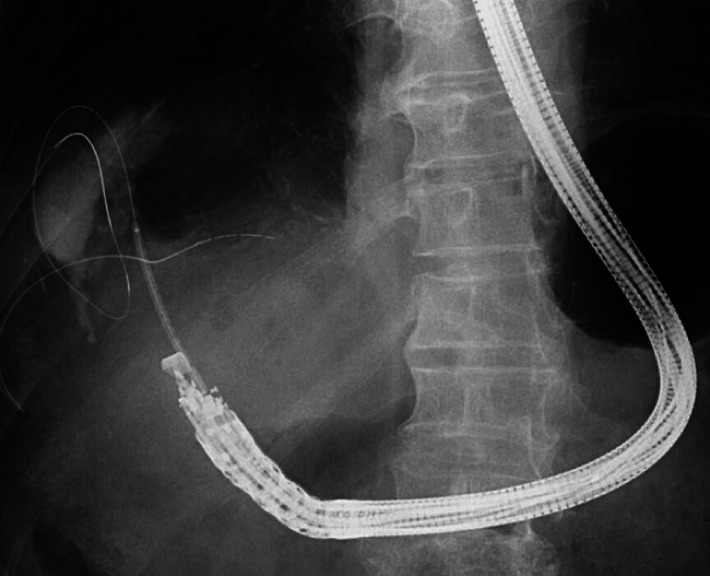
**Fig. 3** Tract dilation was performed with a balloon catheter of 3 mm in diameter and 6 cm in length (REN Type IT, Kaneka). The expanded balloon was easily recognized on the fluoroscopic image.

**Fig. 4:**
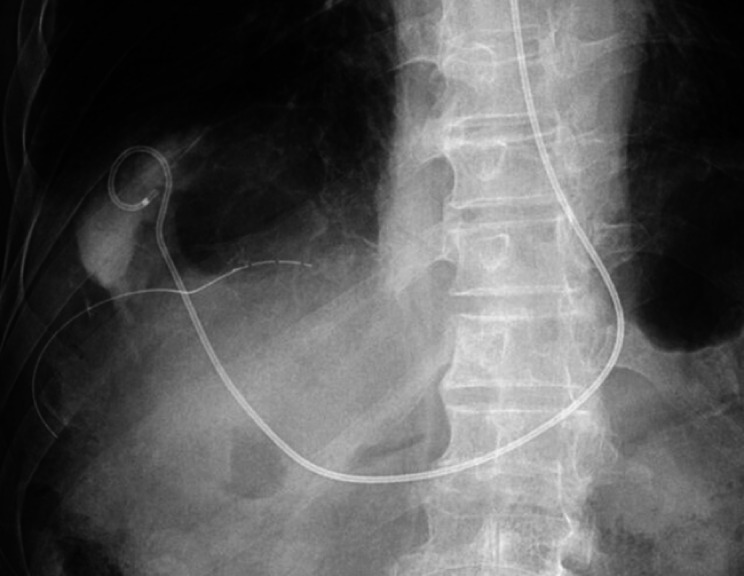
**Fig. 4** A 6-Fr pigtail-type endoscopic nasobiliary drainage (NB-Braid, Piolax) was placed.

Endoscopic ultrasound-guided drainage for infected biloma using a novel long-type balloon catheter.Video 1Video 1

In this case, a conventional-length balloon catheter would require multiple dilations. The use of this long-type balloon catheter allowed simultaneous dilation of the duodenal mucosa and liver parenchyma in a single inflation. This device can simplify tract dilation and shorten procedure time.

Endoscopy_UCTN_Code_TTT_1AS_2AD

## Notes

Endoscopy E-Videos https://eref.thieme.de/e-videos
*E-Videos* is an open access online section of the journal *Endoscopy* , reporting on interesting cases and new techniques in gastroenterological endoscopy. All papers include a high-quality video and are published with a Creative Commons CC-BY license. Endoscopy E-Videos qualify for HINARI discounts and waivers and eligibility is automatically checked during the submission process. We grant 100% waivers to articles whose corresponding authors are based in Group A countries and 50% waivers to those who are based in Group B countries as classified by Research4Life (see: https://www.research4life.org/access/eligibility/ ). This section has its own submission website at https://mc.manuscriptcentral.com/e-videos .
